# Some remarks on peer review and preprints

**DOI:** 10.1590/0074-02760230001

**Published:** 2023-06-26

**Authors:** Adeilton Alves Brandão

**Affiliations:** 1 Fundação Oswaldo Cruz-Fiocruz, Instituto Oswaldo Cruz, Rio de Janeiro, RJ, Brasil

In 2013 the biomedical research community saw the birth of its first preprint server, the bioRxiv (https://www.biorxiv.org/). Since then, we have witnessed both the growing of repositories dedicated to the fast and non-peer-reviewed publication of scientific articles (preprint articles) and the community mind changing about the benefits (and risks!) of the immediate release of research results. It is a new scenario for biomedical and biological researchers but not for the physics research community whose arXiv physics has been publishing preprints since 1991.

In view of current challenges, we may say that scientific publishing is now living under the “disruption of preprints”! For sure, the scientific publishing environment is not the same as the one we saw at the dawn of 21st century. Scientific editors must now think about two things: (i) a new concept of “publishing papers”, and (ii) how to proper (and innovatively) evaluate the contribution these freshly released papers might bring to society.

The origins of the scientific article dates back to XVII century, with the creation of the “Journal de Savants” and the “Philsophical Transactions of the Royal Society”, both in 1665. Scientists sent letters to theses journals communicating their research results, and over the centuries the letters evolved to a standard format that we today call “scientific article”. Although the current scientific article is very different from the first ‘letter’ published by Phil. Trans. Royal. Soc. in 1665, in terms of message and scope it remains essentially the same object: a relatively short account of procedures and datasets based on the ‘scientific method’ that reveal, describe and explain the natural phenomena.

The science dissemination through “article editing and publishing” is now firmly grounded on the ‘*peer review*’, a procedure aimed at correcting and improving the submitted articles by the critical reading from other scientists. It is expected that the external peer reviewing assures the research credibility, the correctness of scientific registry and support from society. The peer review was not practiced by all journals until the second half of twentieth century - for example, Nature Journal is said to start using peer review only at the beginning of 1970s, see Baldwin.^1^ It is assumed that at the turning of 21st century no journal could be taken seriously if the ‘peer review’ was not included in its editorial practice. Therefore, peer review is regarded as the ultimate criterion to consider a manuscript as being finally ‘published’ and ‘validated’ as conveyor of scientific evidence.

Although most papers undergoing peer review get improvements that make them “ready” to public reading, *i. e.*, they become ‘publishable’, the external reviewing does not prevent that “non appropriate” papers reach the end of the scientific publishing pipeline. Nowadays there are so many journals that, in the words of our former editor Hooman Momen, “...*a researcher does not get published only if she/he does not want to!*”. Therefore the peer review in its current format appears to be reaching a saturation level - too many papers being submitted to journals that get less and less expert researchers in the field to review them. The result is a quality control system that, despite being praised as the “credibility pillar” of all science, does not efficiently work for all published articles. Thus, if standard peer review is not working at full pace, why should it be seen as indispensable in its current format (anonymous, closed, ignoring the power of digital technology)? For the present, there is no surrogate for this checking system! It is the only method scientific editors have to help them pick up relevant papers to their journals and readers.

To cope with this limitation scientific editors should consider the road map provided by the open science. Critical reading, suggestion of improvements and red flagging of manuscripts in open repositories are actions that every scientist should naturally do regardless of formal invitation from an editor for ‘peer reviewing’. Certainly this ‘self-regulation’ act from scientists will reduce the burden on formal peer review system (*e.g.*, journal invited peer review). Of course, a major change should occur in current academic set of reward and incentives for scientists both to publish and to review the work of others researchers.

To help accelerating these changes, scientific editors can do some actions to ameliorate the article publishing and peer review system:

a) Prioritise submissions already available in preprint server;

b) Start practicing the “open and non-anonymous peer reviewing of every submitted article”.

The motivation for the action (a) lies on the fact that immediate release (and reading) of scientific articles has reached a “non-reversible path’, and journals have lost the “monopolistic function” of first hand disseminator of scientific evidence.

Action (b) is obvious from the open science perspective: readers should know who has evaluated that manuscript, what changes reviewers have suggested, which (and why) additional experiments and data they have asked to authors, and how the editor has managed this “peer-to-peer” conversation. In some instances, the conversation among authors, reviewers and editors are as interesting as the article itself! The act of opening the current closed conversation during peer reviewing can be seen as complement to consolidate the immediate release of research results through preprint servers. There is no reason anymore to restrain this conversation from readers if they can also *talk* to authors of an article already in preprint (though there is no evidence that such a conversation is really happening now!).

The actions above work as checkpoints and should help to strength the peer review system as the main pillar of the science credibility as well as improve the research communication!
